# Health disparities in chronic back pain and associated mortality seen in ischemic cardiac disease: a commentary

**DOI:** 10.1186/s13690-021-00710-4

**Published:** 2021-11-09

**Authors:** Ashruta Patel

**Affiliations:** Philadelphia College of Osteopathic Medicine, Georgia Campus, 625 Old Peachtree Road NW, GA 30024 Suwanee, USA

**Keywords:** Coronary artery disease, Ischemic heart disease, Chronic pain, Back pain

## Abstract

Prescription opioid use for nonmalignant chronic pain has grown in the US over the last decade. Those with chronic back pain have a higher risk of mortality from ischemic heart disease than those without. Studies have demonstrated a higher prevalence of cardiac disease in adults who report chronic pain. In addition, there is research that supports some association with pain sites and cardiovascular morbidity. Studies have also shown a high-grade chronic neck pain to be more associated with cardiovascular conditions when compared to moderate or low-grade chronic pain. Given this information, it is important to assess pain medication burden present in those who have a diagnosis of coronary artery disease and chronic lower back pain.

## Background

There are several diseases that have been associated with lower back pain, including heart disease, the number one cause of mortality in the US [1]. Some studies report coronary heart disease showing a relationship with back pain severity and death rate [2]. In addition, chronic musculoskeletal [3, 4] and diffuse pain have been associated with coronary artery disease [5, 6]. One study found those with sedentary lifestyle and classified within metabolic syndrome to have higher likelihood of developing nonspecific low back pain [7]. Chronic pain may be associated with increased risk of high blood pressure [8]. Pain that is uncontrolled may affect the sympathetic autonomic nervous system to release hormones that may cause stress on the cardiovascular system resulting in complications, such as elevated blood pressure, chest pain, or coronary spasms. Given results from previous research studies, we anticipate there to be a greater burden of pain medicine use in those who have a diagnosis of coronary artery disease and lower back pain.

## Interventions

Implementing interventions within a hospital/clinic setting to study the association between a cohort of patients with coronary artery disease and chronic lower back pain when compared to people without any coronary artery disease, and the burden chronic pain medication use, has the potential to identify strategies to reduce opioid abuse. For example, using the electronic medical record to identify a subset of adults with heart disease who present to a chronic pain management clinic with symptoms of lower back pain and are on specific medication therapy (opioid burden). Factors such as, race/ethnicity, gender, education, income, disability, geography, sexual orientation, poor access to health care, and inequalities can play a major impact on health outcomes, health-related decision making, quality of life, and unnecessary health-care costs. It is important to evaluate whether or not disparities exist in those who use opioids and have coronary artery disease, to help address whether or not this group may have a higher burden of pain medication use and if this patient population may require alternative pain management options. Although patients have a diagnosis of pain, pain scores are patient-dependent and one may interpret specific symptoms differently than others, making the clinical decision to begin certain pain medications to be varied among various providers, particularly when patients present to clinic on several different regimens for pain control.

## Conclusions

Implementing hospital/clinic-based interventions to determine the opioid burden in those who have chronic pain and coronary artery disease can help address strategies necessary to reduce opioid abuse in vulnerable patients who face several barriers to their care and are also high risk for mortality.

## Data Availability

Not applicable.
